# Hybrid Wheat Prediction Using Genomic, Pedigree, and Environmental Covariables Interaction Models

**DOI:** 10.3835/plantgenome2018.07.0051

**Published:** 2019-03-01

**Authors:** Bhoja Raj Basnet, Jose Crossa, Susanne Dreisigacker, Paulino Pérez‐Rodríguez, Yann Manes, Ravi P. Singh, Umesh R. Rosyara, Fatima Camarillo‐Castillo, Mercedes Murua

**Affiliations:** ^1^ International Maize and Wheat Improvement Center (CIMMYT) Apdo. Postal 6‐641 06600 Mexico City Mexico; ^2^ Colegio de Postgraduados CP 56230, Montecillos Edo. de México México; ^3^ Syngenta Seeds 12, Chemin de l'Hobit 31790 Saint‐Sauveur France; ^4^ Syngenta Seeds 11055 Wayzata Boulevard Minnetonka MN 55305

## Abstract

In this study, we used genotype × environment interactions (G×E) models for hybrid prediction, where similarity between lines was assessed by pedigree and molecular markers, and similarity between environments was accounted for by environmental covariables. We use five genomic and pedigree models (M1–M5) under four cross‐validation (CV) schemes: prediction of hybrids when the training set (i) includes hybrids of all males and females evaluated only in some environments (T2FM), (ii) excludes all progenies from a randomly selected male (T1M), (iii) includes all progenies from 20% randomly selected females in combination with all males (T1F), and (iv) includes one randomly selected male plus 40% randomly selected females that were crossed with it (T0FM). Models were tested on a total of 1888 wheat (*Triticum aestivum* L.) hybrids including 18 males and 667 females in three consecutive years. For grain yield, the most complex model (M5) under T2FM had slightly higher prediction accuracy than the less complex model. For T1F, the prediction accuracy of hybrids for grain yield and other traits of the most complete model was 0.50 to 0.55. For T1M, Model M3 exhibited high prediction accuracies for flowering traits (0.71), whereas the more complex model (M5) demonstrated high accuracy for grain yield (0.5). For T0FM, the prediction accuracy for grain yield of Model M5 was 0.61. Including genomic and pedigree gave relatively high prediction accuracy even when both parents were untested. Results show that it is possible to predict unobserved hybrids when modeling genomic general combining ability (GCA) and specific combining ability (SCA) and their interactions with environments.

**Abbreviations**
BLUP: best linear unbiased prediction
CV: cross‐validation
DTF: days to flowering
DTH: days to heading
DTM: days to maturity
GBLUP: genomic best linear unbiased predictor
GCA: general combining ability
GY: grain yield
G×E: genotype × environment interaction
PHT: plant height
SCA: specific combining ability
SNP: single‐nucleotide polymorphism

## Core Ideas

- Genomic prediction of hybrid wheat
- Introducing pedigree and environmental covariables
- Modeling genomic × environment interaction

Hybrid wheat is a promising technology for enhancing global wheat production in the future. Recently, breeding hybrids of self‐pollinated species, such as wheat (*Triticum aestivum* L.), rice (*Oryza sativa* L.), and barley (*Hordeum vulgare* L.), has regained significant interest (Li et al., 2017). In addition to enhancing yield potential, wheat hybrids have more stable performance across various environments, including environments with different types of stress, than traditional pure‐line cultivars (Longin et al., 2013; Mühleisen et al., 2014). To accelerate the genetic gain with limited resources, predicting hybrid performance is of fundamental importance in modern hybrid breeding programs. Historically, best linear unbiased prediction (BLUP) has been useful for predicting the performance of unobserved (in field evaluations) maize (*Zea mays* L.) single crosses using the pedigree relationship (coancestry) of all crosses (observed and unobserved) and the performance of observed crosses (Bernardo, 1994; 1996a,b). When studying and assessing hybrid performance, two sources of variation are important: the estimated additive effects among lines based on the variance of the GCA of the male and female parents ($\sigma_{\text{GCA}}^{2}$), and the dominance and epistatic effects among lines based on the variance of the SCA of the cross between lines ($\sigma_{\text{SCA}}^{2}$). Although different authors have different opinions about the importance of SCA and GCA as the main factors that determine hybrid performance (Bernardo, 1994, 1996a,b; Duvick et al., 2004; van Eeuwijk et al., 2010; Massman et al., 2013), both are fundamental sources for prediction based on either pedigree or genome‐wide marker information of the lines forming the single cross. Extensive multienvironment testing of single crosses allows sampling a wide array of single‐cross × environment interactions, and hybrids unobserved in field evaluation can be predicted based on existing data from other observed hybrids.

The BLUPs of the unobserved hybrids based on the pedigree relationship matrix originally developed by Bernardo (1994; 1996a,b) are analogous to the prediction of unobserved hybrids using dense molecular markers. The genomic‐enabled prediction of unobserved hybrids was first proposed by Piepho (2009). Genomic prediction studies conducted over the last decade include BLUPs based on different prediction methods such as ridge regression and the genomic best linear unbiased predictor (GBLUP) (VanRaden, 2008). These prediction methods have been extensively employed in maize (Xu et al., 2014; Lehermeier et al., 2014; Schrag et al., 2010; Technow and Melchinger, 2013; Technow et al., 2012, 2014; Acosta‐Pech et al., 2017).

Unlike conventional wheat breeding (inbred‐line development following two‐way or three‐way crosses and release), hybrid wheat breeding has been suggested as a method to increase grain yield performance by exploiting heterosis. Researchers have found that midparent grain yield performance is moderately associated with hybrid wheat performance (Longin et al., 2013), thereby imposing the need to establish extensive field evaluations. This makes the prediction of hybrid wheat based on pedigree and genome‐wide markers of the parental lines an important strategy, where phenotyping of a representative set of hybrids remains a fundamental step for the prediction of unobserved hybrids.

Several recent studies have shown different hybrid wheat genomic‐enabled prediction accuracies. For example, Zhao et al. (2013) used data from a wheat breeding program and made genomic‐enabled predictions of unobserved hybrids using five prediction models under additive (GCA) and dominance (SCA) effects. They found moderate to high prediction accuracy for all five models, although adding the dominance component did not increase genomic prediction accuracy. In agreement with the poor contribution of dominance to heterosis for grain yield, Jiang et al. (2017) demonstrated the fundamental role that epistatic effects play in grain yield heterosis using a set of 1604 hybrids and their 135 parental wheat lines evaluated in 11 environments. The authors found that the hybrids' performance surpassed the performance of midparent heterosis on average by 10%. Similarly, promising genomic‐enabled prediction accuracies were found by Li et al. (2017) when predicting a large number of three‐way barley hybrids using ridge regression BLUP.

As mentioned earlier, evaluating hybrid performance in multienvironment trials for assessing G×E plays an important role in selecting the most productive and stable hybrids. Burgueño et al. (2012) were the first to use marker and pedigree models for assessing G×E under genomic prediction. Jarquín et al. (2014) proposed a model where the main effects and interaction effects of markers and environmental covariables are introduced using highly dimensional random variance–covariance structures of markers and environmental covariables. Incorporating G×E into GBLUP models could increase the accuracy of hybrid performance. However, none of the previous studies has explicitly incorporated G×E into hybrid prediction models.

Acosta‐Pech et al. (2017) were the first to propose an extension of the models of Technow et al. (2012) and Massman et al. (2013) using the genomic G×E model of Jarquín et al. (2014) as a framework with the interactions of SCA effects × environment and GCA effects × environment in a maize hybrid data set that included 2724 maize hybrids from 531 maize inbred lines that were evaluated for 12 yr in 58 different locations. The results indicated that hybrid prediction accuracy increased substantially when G×E interaction was added to the prediction models. On average, genomic models that include GCA × environment interaction and SCA × environment interaction had 12 to 21% higher predictive ability than genomic models without interaction. The authors concluded that including G×E in the prediction of unobserved maize hybrids increases prediction accuracy.

Most genomic hybrid prediction studies ignore G×E and do not include environmental covariables model similarities between environments. In maize, Acosta‐Pech et al. (2017) only used marker information but not pedigree information or environmental covariables to predict hybrid performance. Therefore, the main objectives of the present study were (i) to study genomic‐enabled prediction of large sets of single‐cross wheat hybrids using models with various combinations of pedigree, markers, and their interaction with environments; and (ii) to investigate the prediction accuracy of these models under four CV schemes: prediction of hybrids when (i) both observed male and female parents are included in the training set (T2MF), (ii) all observed male parents except one are included in the training set (T1M), (iii) some observed female parents are included in the training set (T1F), and (iv) only one male and a certain percentage of the observed females are included in the training set.

## MATERIALS AND METHODS

### Phenotypic Data

**Fig. 1:**
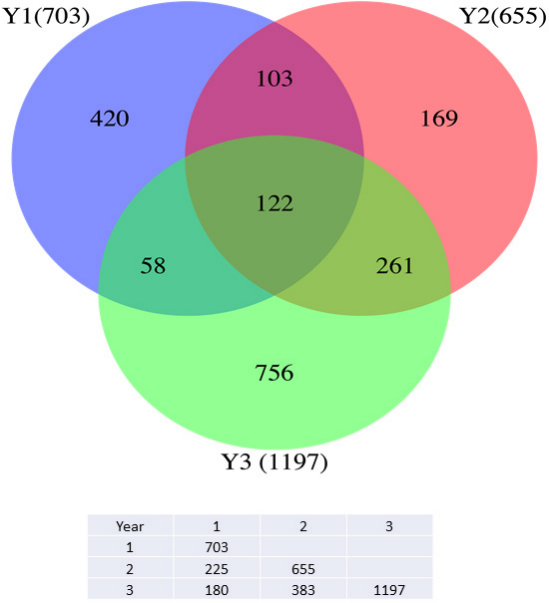
Venn–Euler diagram with the number of hybrids tested in 3 yr at CIMMYT CENEB near Ciudad Obregon, Mexico. The numbers in parentheses represent the actual number of hybrids (H) with single nucleotide polymorphism genotypes that were used for genomic prediction during Year 1 (2014–2015), Year 2 (2015–2016), and Year 3 (2016–2017).

A total of 1888 experimental hybrids obtained by crossing 18 males and 667 females were evaluated in field trials for 3 yr at CIMMYT's Campo Experimental Norman E. Borlaug (CENEB or the Norman E. Borlaug Experiment Station) near Ciudad Obregon, Sonora, Mexico. In particular, 703, 655, and 1197 hybrids were evaluated during the winter growing seasons in 2014 to 2015 (Year 1), 2015 to 2016 (Year 2), and 2016 to 2017 (Year 3), respectively, with 225 and 383 common hybrids in each consecutive year (Fig. 1). The elite male and female parents were chosen from CIMMYT's spring bread wheat program based on their per se performance for the traits of interest, suitability for producing hybrids, and ancestral diversity measured with a coefficient of parentage. The hybrids were produced using a chemical hybridizing agent, provided by Syngenta Inc., in alternate male and female strip plots measuring 6.4 m^2^.

Hybrids and parents were evaluated in 12 by 10 (Year 1) and 10 by 20 (Years 2 and 3) α‐lattice trials with two replications. To establish uniform plant density, 1000 seeds were sown in each 4.8‐m^2^ yield trial plot. The trials were conducted in a high‐yield‐potential environment with four supplementary irrigations and using standard agronomic practices. The set of two advanced checks and all the male parents present in the hybrids being tested in a particular year were planted in all trials. The female parents and hybrids were planted side by side in all the trials. Days to heading (DTH), days to flowering (DTF), days to maturity (DTM), plant height (PHT), and grain yield (GY) per plot were recorded for each entry.

Phenotypic data were analyzed by using a mixed linear model implemented in META‐R (Alvarado et al., 2015) software, where genomic best linear unbiased predictors were estimated after fitting the model with genotype, trial, and replication nested within trials, and sub‐blocks nested within trials and replications. Best linear unbiased estimates were obtained for each hybrid and parent and used for further analyses. Broad‐sense heritability (*H* ^2^) based on entry means within trials was estimated as follows: $H^{2}=\sigma_{g}^{2}/\left[\sigma_{g}^{2}+\sigma_{e}^{2}/r\right]$, where $\sigma_{g}^{2}$ and $\sigma_{e}^{2}$ are the genotypic variance and variance resulting from experimental error, respectively, and *r* is the number of replicates (Holland et al., 2003).

### Genotypic Data

The 18 male and 667 female parents were genotyped using the Illumina iSelect 90K Infinitum SNP genotyping array in the first year and the Illumina Infinium 15K wheat SNP array (TraitGenetics GmbH) in the second and third years. A total of 13,005 single‐nucleotide polymorphisms (SNPs) remained after combining the three datasets. Markers with <15% missing values were kept; then, after cleaning from missing, the remaining missing markers were imputed using observed allele frequencies using the naïve method. After imputation, markers with <0.05 minor allele frequency were removed. A total of 10,250 markers were used for further analysis. Although a larger set of hybrids and parents was evaluated in the field, only hybrids derived from SNP‐genotyped parents were used for genomic predictions and different numbers of hybrids were included in each year (Fig. 1).

### Environmental Covariables

The environmental covariables used to model genomic G×E and pedigree G×E are provided as Supplemental Material (provided in the online version of this manuscript) and were collected for each growing cycle (Years 2014–2015, 2015–2016, and 2016–2017) extending from mid‐December to mid‐May of the following year. The growth cycle was divided into 12 10‐d periods to group the maximum, minimum, and average temperatures; precipitation; and growing degree days. The mean values of each 10‐d period were used for each environmental covariable.

### Statistical Models

The statistical models included in this study are based on genomic relationship matrices derived from markers (Acosta‐Pech et al., 2017). Here we extended the models to include a marker‐based genomic relationship matrix for parents and hybrids and a pedigree‐based relationship matrix. In addition, we incorporated into the model the environmental covariables collected during the 3 yr. In the following sections, we provide brief descriptions of the five models, including genomic and pedigree information for GCA (male and female) and SCA (hybrid), and their corresponding interactions with the environmental covariables. We also describe four types of prediction problems that we investigated for comparing the genomic prediction accuracy of five models.

#### Genomic Main Effect Model: GBLUP + Env model (M1)

The linear model for single‐cross performance that includes the environment effect is given as follows (Technow et al., 2014):

(1) $$y=Z_{E}\beta_{E}+Z_{M}g_{M}+Z_{F}g_{F}+Z_{H}h+\varepsilon$$

where **y** is the response vector (i.e., the hybrids' adjusted phenotypic information); **Z**_E_ is the design matrix for environments (year); **β**_E_ is the vector of environmental effects, $\beta_{E}∼N\left(0,\sigma_{E}^{2}I\right)$; **g**_M_ is the vector of random effects due to the GCA of markers for paternal lines (males, M); **g**_F_ is the vector of random effects due to the GCA of markers for maternal lines (females, F); and **h** is the vector of SCA random effects for the crosses (hybrids, H). The incidence matrices **Z**_M_, **Z**_F_, and **Z**_H_ relate **y** to **g**_M_, **g**_F_, and **h** with $g_{M}∼N\left(0,\sigma_{M}^{2}G_{M}\right)$, $g_{F}∼N\left(0,\sigma_{F}^{2}G_{F}\right)$, and $h∼N\left(0,\sigma_{H}^{2}H\right)$, where, $\sigma_{M}^{2}$, $\sigma_{F}^{2}$, and $\sigma_{H}^{2}$ are variance components associated with GCA and SGA, and **G**_M_, **G**_F_, and **H** are relationship matrices for parental and maternal lines and hybrids, respectively. Finally, $\varepsilon ∼N\left(0,\sigma_{\varepsilon }^{2}I\right)$, where $\sigma_{\varepsilon }^{2}$ is the variance associated with the residuals.

The relationship matrices **G**_M_ and **G**_F_ were computed using markers (VanRaden, 2008). Let **X**_*m*_, *m* ∈ {Male, Female} be the matrix of markers and let **W**_*m*_ be the matrix of centered and standardized markers. Then $G_{M}=W_{m}W_{m}^{\prime }/p$ (Technow et al., 2014; López‐Cruz et al., 2015), where *p* is the number of markers. This gives an average diagonal **G**_*m*_ value of ∼1; therefore, $\sigma_{m}^{2}$ is defined on the same scale as $\sigma_{\varepsilon }^{2}$.

The elements of matrix **H** can be obtained directly from matrices **G**_M_ and **G**_F_ (see Bernardo, 2002, pages 231–232; and Technow et al., 2014). In compact notation, matrix **H** for all possible crosses is obtained as the Kronecker product of **G**_M_ and **G**_F_, that is, $H=G_{M}⊗G_{F}$ (e.g., Covarrubias‐Pazaran, 2016).

#### Genomic Main Effect and Interaction Model: GBLUP + Env + Hybrid × Env + Parents × Env model (M2)

Modeling the interaction between markers and environmental covariates can be a very difficult task because of the high dimensionality of the matrix of markers, environmental covariates, or both. Jarquín et al. (2014) suggested modeling this interaction using Gaussian processes, where the associated variance–covariance matrix induces a reaction norm model. The study showed that assuming (i) normality for all the terms involved in the interaction and also assuming that (ii) the interaction is obtained using a first‐order multiplicative model is distributed normally, then the covariance function is the cell‐by‐cell product (Hadamard) of two covariance structures: one describing the genetic information and the other describing the environmental effects. Using this approach, we extended model (1) to include genotype (hybrid) × environment interaction and parent (male and female) × environment interaction. The model is as follows:

(2) $$\begin{array}{c} y=Z_{E}\beta_{E}+Z_{M}g_{M}+Z_{F}g_{F}+\\ Z_{H}h+u_{H}+u_{M}+u_{F}+\varepsilon \end{array}$$

where $u_{H}∼N\left(0,\sigma_{\text{HE}}^{2}V_{H}\right)$, $u_{M}∼N\left(0,\sigma_{\text{ME}}^{2}V_{M}\right)$, $u_{F}∼N\left(0,\sigma_{\text{FE}}^{2}V_{F}\right)$; $\sigma_{\text{HE}}^{2}$, $\sigma_{\text{ME}}^{2}$, and $\sigma_{\text{FE}}^{2}$ are variance components associated with hybrid, female × environment, and male × environment interactions, respectively; and **V**_H_, **V**_M_, and **V**_F_ are their associated variance–covariance matrices. The variance–covariance matrix is given by $V_{H}=Z_{H}H{Z^{\prime }}_{H}\#Z_{E}\Omega {Z^{\prime }}_{E}$, where # stands for the Hadamard product and **Ω** is a relationship between environments computed using the environmental covariables (Jarquín et al., 2014). Note that if observations are sorted by environment, then **V** is a block‐diagonal matrix whose structure is similar to the Marker × Environment model of López Cruz et al. (2015). The variance–covariance matrices **V**_M_ and **V**_F_ can be similarly derived and are as follows: $V_{M}=Z_{M}G_{M}Z_{M}^{\prime }\#Z_{E}\Omega Z_{E}^{\prime }$ and $V_{F}=Z_{F}G_{F}Z_{F}^{\prime }\#Z_{E}\Omega Z_{E}^{\prime }$ (see Jarquín et al., 2014, for more details).

#### Pedigree Main Effect Model: Pedigree + Env Model (M3)

This model is similar to model (1), but the relationships between individuals were built using the additive relationship matrix derived from pedigree.

#### Pedigree Main Effect and Interaction Model: Pedigree + Env + Hybrid × Env + Parents × Env model (M4)

This model is similar to model (2), but the relationship between individuals was built using the additive relationship matrix derived from pedigree.

#### Genomic and Pedigree Main Effect and Interaction Model: GBLUP + Pedigree + Env + Hybrid × Env + Parents × Env Model (M5)

This complete model includes marker and pedigree information, as well as all the main effects and interactions with environmental covariables in the same model.

### Software and Computing

The models described above were fit using Bayesian methods implemented in the BGLR statistical package implemented in R (Pérez‐Rodríguez and de los Campos, 2014). Inferences were based on 10,000 Gibbs sampler iterations, and the first 5000 were discarded. Variance components were estimated using all available records. The prediction accuracy (*r*_G_) was estimated as Pearson's correlations between observed and predicted values of individuals.

### Scripts for Fitting the Models using BGLR

Supplemental material (included with the online version of this manuscript) contains the R code for fitting the models used in this study. The models were fitted using the BGLR statistical package (Pérez‐Rodríguez and de los Campos, 2014) and use the same computational approach described in Acosta‐Pech et al. (2017). To speed up computations in BGLR, we use the eigenvalue decomposition of the variance–covariance matrices in all models.

### Description of Prediction Problems using Various Cross‐Validation Strategies

We studied several genomic prediction problems such as the genomic prediction of hybrids (for crossing tested males and tested females) evaluated in some years but not in others or prediction of hybrids formed by crossing tested males with untested females or prediction of hybrids formed by crossing untested males with tested females or prediction of hybrids formed from crossing untested males and untested female. We provided results for these prediction problems by using different cross‐validation schemes. The four strategies used were four cross‐validation schemes (T2MF, T1F, T1M, and T0MF) at two levels of males and females (tested and untested). Thus, T2MF is the prediction of hybrids obtained by crossing tested (observed training set) males and tested (observed training set) females; T1F is the prediction of hybrids obtained by crossing untested males with tested female; T1M is the prediction of hybrids obtained by crossing tested males with untested female; and T0MF is the prediction of hybrids formed by crossing untested males and females.

### Cross‐Validation Schemes

**Fig. 2:**
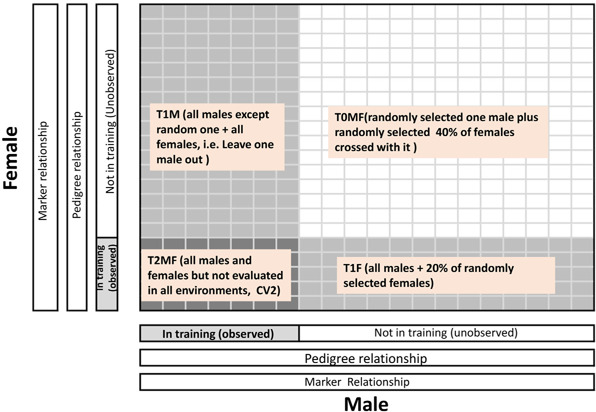
Four prediction problems of tested and untested males and females. T2MF, single crosses of tested males and females using fivefold random cross‐validation with 80% of data in the training set and 20% in the testing set; T1M, leave one tested male out and predict the single cross with untested females; T1F, single crosses of tested females with untested males using fivefold random cross‐validation with 80% of data in the training set and 20% in the testing set; T0MF, single crosses predicted using untested males and females.

The four types of prediction problems we assessed are graphically displayed in Fig. 2. Below we describe the four cross‐validation schemes.

#### Cross‐Validation T2FM

To test the prediction ability of the proposed models, we assessed a common problem breeders face when testing new germplasm in incomplete field trials, that is, predicting the performance of hybrids in a set of environments (sparse testing). All the hybrids and their male and female parents are in the training set (Fig. 2), that is, they were observed in the field. The hybrids had been evaluated in some environments but not in others, and their performance had to be predicted in environments (e.g., years) where they had not been evaluated. To address this problem, we performed a random cross‐validation analysis using a scheme that is known as cross‐validation CV2 (Burgueño et al., 2012), which considers some hybrids that were observed in some environments while missing in others. In CV2, the individual plot records are assigned to folds, so that a hybrid's individual records are potentially assigned to different folds (Jarquín et al., 2014). In T2FM, we assume that the hybrids obtained by crossing both parents have been evaluated.

Figure 2 shows that the aim is to predict those lines that appear in the bottom left panel (T2MF [CV2]) having observed the performance of these males crossed with other observed females, other unobserved males crossed with these same females, and other observed males crossed with other unobserved females. The hybrids were assigned randomly to five folds, and each fold was predicted using the remaining four folds in a proportion of 20 to 80% (20% of the phenotypic records where predicted using the remaining 80%).

#### Cross‐Validations T1M and T1F

Additionally, we performed a fivefold cross‐validation assigning females to folds; therefore, if a set of females is assigned to the training–testing, set, then all the corresponding hybrids obtained when making crosses are also assigned to the training–testing, set. The training set includes all the hybrids obtained when using 80% of the females crossed with the males and predicting the remaining 20% of the females crossed with the males, that is, the goal in T1F is to predict the performance of these hybrids (Fig. 2). We also performed a cross‐validation that assigns males to folds. In this scheme, T1M, the training set, includes all the hybrids obtained when crossing 17 males with the females and the testing set consists of predicting the crosses between one male and the females, that is, the goal here is to predict the crosses of one male at a time (leave one male out, Fig. 2). In both cases, we constrained the prediction only to the available number of hybrids with phenotypical records.

As previously described, the random T1F scheme mimics the problem of predicting hybrid performance using males whose crosses with any of the tested females were not observed. Figure 2 depicts the prediction of untested males crossed with those tested females and no information about these males was observed in the other females since these were masked as missing. The genetic similarities between males in training–testing sets play an important role in the prediction accuracy of the models. Females were assigned randomly to folds (five folds; with 20% of the lines being predicted using the phenotypic information of the remaining 80% of the genotypes) such that all the phenotypes from the same male appear in the same fold; then each fold was predicted using the remaining four folds, one at a time.

Cross‐validation T1M aims to predict hybrid performance of the observed males with new (untested) females. The idea is to predict those males observed in crosses with females, but there are no data about any male tested before with those females. This method is used when there are no phenotypic records of any male being crossed with females and the success of predictive ability will depend partly on whether those females are related to the females in the training set as well as on the performance and number of phenotypic records of the same genotypes in the testing set observed with other females. Here the predictions are made leaving one male out and using the remaining males as the training set. Then the correlations between the predicted and observed values for each male are computed. This procedure does not involve random partitioning; thus it is implemented only once (leave one male out).

#### Cross‐Validation T0MF

For any given male, we predict all the hybrids that are obtained when crossing that specific male with 40% of the females selected at random (predicting 60% of the crosses). This is the most difficult random‐cross‐validation prediction problem because none of the parents of the hybrids have been previously evaluated in the field, that is, the parents' GCA and SCA remain unknown. However, prediction can be achieved by borrowing information from pedigree and genomic relationships between males and females (Fig. 2), as well as from correlated environments expressed in the environmental relationship matrix. In these cross‐validations, hybrids are predicted based on males and females that were never observed. The only source of information comes from different unobserved (not in training) males observed in crosses with other unobserved (not in training) sets of females. In practical situations, this is the case of the prediction of new hybrids that were made by crossing new unobserved males with new unobserved females. The prediction accuracy will depend on the genetic similarities among males and females in the training and testing set.

### Availability of Data and Supplemental Materials

We have uploaded the phenotypic, genotypic (G), and pedigree (A) data used in this study as well as Supplemental Material–Variance Components and Supplemental Material–Environmental Covariables (http://hdl.handle.net/11529/10548129).

## RESULTS

**Fig. 3:**
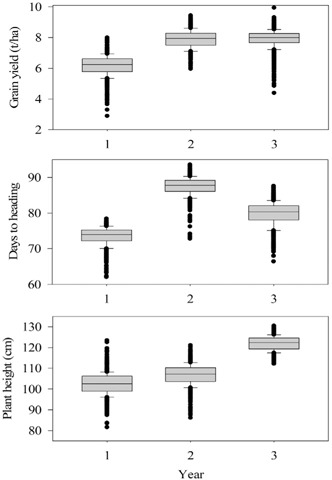
Boxplots of data collected from hybrid trials in Year 1 (2014–2015), Year 2 (2015–2016), and Year 3 (2016–2017) for (a) grain yield (t ha^−1^) (GY); (b) days to heading (days) (DTH); and (c) plant height (cm) (PHT).

**Fig. 4:**
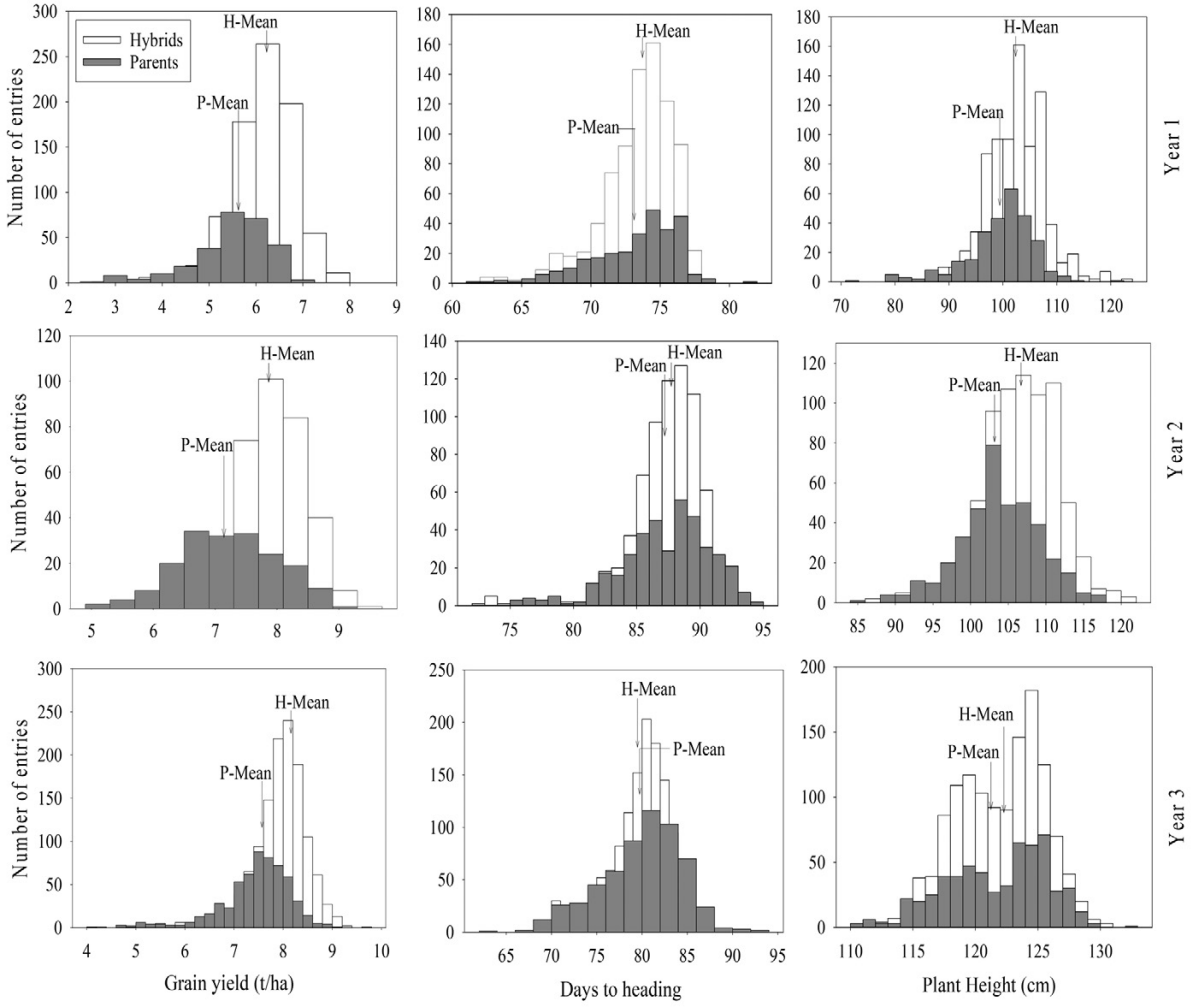
Phenotypic distribution of hybrids and parents for grain yield, days to heading, and plant height for 3 yr: Year 1 (2014–2015), Year 2 (2015–2016), and Year 3 (2016–2017). The phenotypic mean for hybrids (H‐Mean) and parents (P‐Mean) are indicated with arrowheads.

In the field trials, the average grain yield was 6.1, 7.6, and 7.8 t ha^−1^ in Years 1, 2, and 3, respectively (Fig. 3; Table 1). The shortest vegetative growth period was observed in Year 1, with average DTH of 73, followed by Year 3 (average DTH = 80) and Year 2 (average DTH = 87). The plants grew tallest in Year 3 compared with the previous 2 yr. In all the years, heterosis for GY was more pronounced than heterosis for DTH and PHT (Fig. 4). On average, for GY, the hybrids outperformed the parents by 0.43, 0.46, and 0.68 t ha^−1^, in Years 1, 3, and 2, respectively. As such, this was 7.5, 6.2, and 9.5% higher than the parental average for the respective years. The hybrids were slightly taller than the parents, but there was no observable difference in DTH between parents and hybrids (Fig. 4). The broad‐sense heritability (repeatability) estimates were relatively high in all the years, ranging from 0.66 to 0.82 for GY, 0.93 to 0.97 for DTH, and 0.51 to 0.77 for PHT (Table 1). Similarly, high‐quality experiments across years were obtained with low coefficients of variation (CV = 1–8%) for all the traits including GY (Table 1).

**Table 1:** Heritability, grand mean, least significance difference (LSD) at the 0.05 probability level, and coefficient of variation (CV%) of grain yield (GY), days to heading (DTH), days to flowering (DTF), days to maturity (DTM), and plant height (PHT) for hybrid trials established in Cd. Obregon, Mexico, during 3 yr: 2014–1015 (Year 1), 2015–2016 (Year 2) and 2016–2017 (Year 3).

	GY	DTH	DTF	DTM	PHT
	t ha^−1^	d			cm
Year 2014–2015					
Heritability	0.66	0.93	0.93	0.96	0.77
Grand mean	6.10	73.50	77.90	117.4	101.7
LSD(0.05)	0.71	1.17	1.13	0.86	4.11
CV %	8.4	1.20	1.00	0.50	2.9
Year 2015–2016					
Heritability	0.73	0.96	0.95	0.94	0.51
Grand mean	7.6	87.7	91.1	129.1	105.6
LSD(0.05)	0.79	0.99	1.03	0.97	6.10
CV %	5.31	0.81	0.81	0.54	4.17
Year 2016–2017					
Heritability	0.82	0.97	0.97	0.71	0.70
Grand mean	7.8	79.9	83.2	106.0	122.1
LSD(0.05)	0.72	1.60	1.81	6.79	1.91
CV %	4.56	0.99	1.09	3.20	0.78

### Estimating Variance Components of Full Data

Variance components of the fitted complete model (M5) for GY were obtained based on GCA and SCA and their interaction with environments for year (E), genomic (G), and pedigree (A) (Table 2). Estimated variance components of Models M1 through M5 for all the traits are given in tables of Supplemental Material—Variance Components (http://hdl.handle.net/11529/10548129). In Table 2, for most of the models, the year component (environment) accounted for the largest proportion of the variance followed by genomic GCA for males. The small proportion of genetic variance is explained by the SCA, which suggests that dominance variation played a smaller role than additive variation in grain yield heterosis. In general, the G×E components were similar or higher than the SCA and female GCA components. The observations were very similar for GY, DTH, and DTF. However, for DTM and PHT, environmental interactions with parental GCA were higher than the GCA variances per se.

**Table 2:** Variance components of Model M5 for traits grain yield (GY), days to heading (DTH), days to flowering (DTF), days to maturity (DTM), and plant height (PHT) with the main effect of year, genomic (G) and pedigree (A) of females (F), males (M), and hybrids (H), and their interaction with environments (G×E) and (A×E) and their corresponding SD and the percentage of total variance explained by each component.

Variance component	GY			DTH			DTF			DTM			PHT		
	Estimate	SD	Percentage	Estimate	SD	Percentage	Estimate	SD	Percentage	Estimate	SD	Percentage	Estimate	SD	Percentage
	t ha^−1^		%	t ha^−1^		%	t ha^−1^		%	t ha^−1^		%	t ha^−1^		%
Year $\sigma_{E}^{2}$	0.469	0.397	38.317	16.775	16.791	42.979	14.172	9.518	39.927	39.447	27.749	48.211	39.823	26.559	45.056
^F_G^ $\sigma_{F}^{2}$	0.059	0.011	4.820	5.14	0.834	13.169	4.934	0.773	13.901	2.106	0.488	2.574	3.693	0.903	4.178
^M_G^ $\sigma_{M}^{2}$	0.125	0.069	10.212	2.127	1.017	5.450	1.835	0.974	5.170	4.687	1.973	5.728	4.572	2.007	5.173
^H_G^ $\sigma_{H}^{2}$	0.023	0.005	1.879	0.359	0.06	0.920	0.335	0.062	0.944	0.885	0.171	1.082	1.274	0.222	1.441
H_G×E $\sigma_{\text{HE}}^{2}$	0.025	0.005	2.042	0.452	0.071	1.158	0.431	0.069	1.214	1.327	0.233	1.622	2.541	0.544	2.875
F_G×E $\sigma_{\text{FE}}^{2}$	0.034	0.009	2.778	0.733	0.15	1.878	0.689	0.146	1.941	3.224	0.66	3.940	4.757	1.154	5.382
M_G×E (ME)	0.077	0.040	6.291	1.918	0.795	4.914	1.835	0.743	5.170	5.242	2.187	6.407	5.196	2.163	5.879
^F_A^ ${\ddot{\sigma }}_{F}^{2}$	0.035	0.008	2.859	4.767	0.808	12.213	4.594	0.672	12.943	3.834	0.656	4.686	3.274	0.757	3.704
^M_A^ ${\ddot{\sigma }}_{M}^{2}$	0.085	0.044	6.944	2.004	0.953	5.134	1.914	0.927	5.392	6.648	3.984	8.125	4.781	2.177	5.409
^H_A^ ${\ddot{\sigma }}_{H}^{2}$	0.022	0.006	1.797	0.456	0.08	1.168	0.413	0.087	1.164	1.023	0.183	1.250	1.131	0.243	1.280
H_A×E ${\ddot{\sigma }}_{\text{HE}}^{2}$	0.028	0.007	2.288	0.534	0.098	1.368	0.558	0.096	1.572	1.332	0.275	1.628	2.129	0.484	2.409
F_A×E ${\ddot{\sigma }}_{\text{FE}}^{2}$	0.062	0.015	5.065	0.768	0.163	1.968	0.736	0.161	2.074	3.33	0.722	4.070	3.625	0.891	4.101
M_A×E ${\ddot{\sigma }}_{\text{ME}}^{2}$	0.058	0.027	4.739	1.732	0.742	4.437	1.762	0.848	4.964	5.492	2.601	6.712	4.873	2.368	5.513
Residual $\sigma_{\varepsilon }^{2}$	0.122	0.006	9.967	1.266	0.074	3.244	1.287	0.072	3.626	3.244	0.194	3.965	6.716	0.351	7.599

In general, for trait GY in Model M5, the variance component for year explained the largest proportion of the total variance, 38.31%; whereas M_G explained a sizeable portion of the total variance (10.21%) followed by the male genomic interaction term M_G×E, which accounted for 6.29%, indicating that interaction is an important component (Table 2). The variance of male M_A resulting from pedigree additive effects explained 6.94% of the total variance, whereas the interaction terms M_A×E and F_A×E explained a sizeable portion of the total variance (4.73% and 5.06%, respectively). These results indicate that models with interactions may have a little higher genomic‐enabled prediction than those without interactions. Also, genomic male contributions surpassed the size of the variance of pedigree male contributions.

Variance components for all the traits and all five models can be observed in Supplemental Material—Variance Components (http://hdl.handle.net/11529/10548129), where for GY the largest variance component for M1 is M_G (0.255); for M2, the variance components for M_G and M_G×E were the highest (0.1013 and 0.1647, respectively) and similar for M3 and M4 models with high value for the variance of M_A.

### Prediction Accuracy of T2FM

The prediction accuracies of the five models for the hybrids obtained by crossing the observed (tested) males and females (T2FM) are shown in Table 3. For GY, models including genomic G×E (Model M2) with environmental covariables or pedigree G×E (Model M4) with environmental covariables gave higher prediction accuracies (0.64 and 0.64) than those without genomic G×E or pedigree G×E (Model M1, 0.62; Model M3, 0.61). The model including both genomic G×E and pedigree G×E with environmental covariables for GCA of males and females and their single‐cross SCA gave the highest prediction accuracy (0.65) for trait GY. Very similar trends in prediction accuracy were observed for the other traits (DTH, DTF, DTM, and PHT), where including the genomic, pedigree, and interaction with environmental covariables gave higher prediction accuracy. Overall, the highest prediction accuracies were observed for DTH and the lowest for PHT.

**Table 3:** Genomic‐enabled prediction accuracy of five models (1–5) (correlations between observed and predicted values) from random cross‐validation T2FM for grain yield (GY), days to heading (DTH), days to flowering (DTF), days to maturity (DTM), and plant height (PHT). Results of the best predictive model are in boldface and its corresponding SDs are in parentheses.

	Model				
	M1	M2	M3	M4	M5
GY	0.62 (0.04)	0.64 (0.03)	0.61 (0.04)	0.64 (0.04)	0.65 (0.04)
DTH	0.80 (0.02)	0.81 (0.02)	0.80 (0.02)	0.82 (0.01)	0.82 (0.02)
DTF	0.79 (0.02)	0.80 (0.02)	0.80 (0.02)	0.81 (0.02)	0.81 (0.02)
DTM	0.52 (0.05)	0.63 (0.03)	0.54 (0.05)	0.66 (0.04)	0.66 (0.03)
PHT	0.47 (0.05)	0.55 (0.04)	0.45 (0.04)	0.53 (0.04)	0.55 (0.04)

### Prediction Accuracy of T1F

The prediction accuracies of the five models are shown in Table 4. For GY, Models M2 and M4 with genomic G×E with environmental covariables and pedigree G×E with environmental covariables, respectively, gave higher prediction accuracy (Model M2, 0.54; Model M4, 0.52) than Model M1 (0.53) and Model M3 (0.49) without interaction. Model M5 including genomic and pedigree main effects and their corresponding interactions with environmental covariables gave the highest prediction accuracy for GY (0.55). Similar trends in prediction accuracy were found for traits DTH, DTF, DTM, and PHT, where Model M5 with all main effects and interactions gave the highest prediction accuracies (0.52, 0.50, 0.45, and 0.38). Models M2 and M4 with genomic G×E and pedigree G×E also achieved higher prediction accuracies than Models M1 and M3 without interaction (Table 4). Overall, the highest prediction accuracy was observed for grain yield.

**Table 4:** Genomic‐enabled prediction accuracy of five models (1–5) (correlations between observed and predicted values) from random cross‐validation T1F for grain yield (GY), days to heading (DTH), days to flowering (DTF), days to maturity (DTM), and plant height (PHT). Results of the best predictive model are in boldface and SDs are in parentheses.

	Model				
	M1	M2	M3	M4	M5
GY	0.53 (0.08)	0.54 (0.08)	0.49 (0.06)	0.52 (0.05)	0.55 (0.07)
DTH	0.49 (0.07)	0.49 (0.06)	0.46 (0.06)	0.47 (0.08)	0.52 (0.06)
DTF	0.47 (0.07)	0.47 (0.07)	0.46 (0.05)	0.47 (0.06)	0.50 (0.06)
DTM	0.34 (0.05)	0.40 (0.06)	0.39 (0.06)	0.44 (0.07)	0.45 (0.08)
PHT	0.32 (0.04)	0.37 (0.06)	0.30 (0.06)	0.34 (0.06)	0.38 (0.06)

### Prediction Accuracy of T1M

For all traits evaluated, the average prediction accuracies of hybrids obtained by crossing tested males with untested females are shown in Table 5 and Supplemental Table S1–S5 (http://hdl.handle.net/11529/10548129) for each of the 16 available males. On average, the best predictive model across all 16 males was Model M5 with a 0.51 correlation between predicted and observed phenotypic values for tested males (Table 5). Supplemental Table S1 shows eight males that were predicted by Model M5 with accuracies ranging between 0.6025 and 0.7433 for GY. Hybrids from untested females and tested males were well predicted for trait PHT by Model M5 (0.6), whereas for DTH and DTF, Model M3 with pedigree as the main effect and without G×E interaction predicted the untested males with average accuracies of 0.71 and 0.70, respectively.

**Table 5:** Average genomic‐enabled prediction accuracy of five models (1–5) (correlations between observed and predicted values) for 16 males from leave‐one‐male out, T1M for grain yield (GY), days to heading (DTH), days to flowering (DTF), days to maturity (DTM) and plant height (PHT). Results of the best predictive model are in boldface and SDs are in parentheses.

	Model				
	M1	M2	M3	M4	M5
GY	0.39 (0.29)	0.50 (0.19)	0.48 (0.15)	0.50 (0.20)	0.51 (0.19)
DTH	0.70 (0.37)	0.68 (0.40)	0.71 (0.36)	0.69 (040)	0.68 (0.39)
DTF	0.69 (0.40)	0.68 (0.39)	0.70 (0.37)	0.60 (0.38)	0.69 (0.40)
DTM	0.41 (0.38)	0.46 (0.44)	0.47 (0.28)	0.54 (0.31)	0.49 (0.40)
PHT	0.42 (0.21)	0.58 (0.21)	0.39 (0.24)	0.58 (0.12)	0.60 (0.17)

### Prediction Accuracy of T0FM

The most difficult prediction problem involves hybrids obtained by crossing males and females that were never observed (Table 6). For all traits evaluated, the prediction accuracies of the 18 untested males are shown in Supplemental Table S6–S10 (http://hdl.handle.net/11529/10548129). For GY, the best predictive model, on average, across all 16 males was Model M5 with an accuracy of 0.61. Interestingly, for GY (Supplemental Table S6), we found that 14 males in crosses with untested females were predicted by Model M5 with accuracies ranging between 0.5059 and 0.9428. Hybrids from untested males and females were predicted for trait PHT using Model M5 with an accuracy of 0.56. For traits DTH and DTF, Model M2 with genomic G×E interaction using environmental covariables predicted the crosses of untested males and females with average accuracies of 0.70 and 0.73, respectively.

**Table 6:** Average genomic‐enabled prediction accuracy of five models (1–5) (correlations between observed and predicted values) for 16 males from T0FM for grain yield (GY), days to heading (DTH), days to flowering (DTF), days to maturity (DTM), and plant height (PHT). Results of the best predictive model are in boldface and SDs are in parentheses.

	Model				
	M1	M2	M3	M4	M5
GY	0.46 (0.37)	0.60 (0.21)	0.58 (0.22)	0.58 (0.20)	0.61 (0.19)
DTH	0.69 (0.32)	0.70 (0.29)	0.68 (0.35)	0.68 (0.37)	0.69 (0.33)
DTF	0.69 (0.35)	0.73 (0.23)	0.69 (0.36)	0.70 (0.37)	0.70 (0.34)
DTM	0.42 (0.39)	0.41 (0.47)	0.44 (0.40)	0.47 (0.42)	0.45 (0.44)
PTH	0.42 (0.43)	0.54 (0.41)	0.47 (0.26)	0.53 (0.40)	0.56 (0.40)

## DISCUSSION

Hybrid breeding is an efficient system to increase yield potential by breaking the yield plateau in many crops. The CIMMYT Global Wheat Program has reassessed hybrid wheat since 2012 via a public–private partnership with Syngenta International. The CIMMYT elite lines derived from the spring bread wheat breeding program are crossed and the resulting F_1_ hybrids are evaluated primarily in Mexico, with smaller subsets evaluated in India. As we lack distinct male and female heterotic breeding pools, the main challenge is selecting parents for hybrid production. Although the per se performance of inbreeds, plant phenology, and pollination‐related traits are the initial criteria for selecting parents for hybrid production and testing, the number of combinations that can actually be produced and tested in the field is limited. Since the beginning of its hybrid wheat program, CIMMYT has been routinely applying the coefficient of parentage as one of the parental selection criteria used to maximize the genetic distance between the male and female parents. Current studies indicate that pedigree information and molecular marker data can be applied to predict the performance of single‐cross hybrids with or without accounting for G×E interaction. By applying hybrid prediction models, we envision selecting potential hybrid parents from a broader genetic germplasm pool earlier in the breeding cycle after preliminary and elite yield trials of CIMMYT's conventional spring wheat breeding program. This approach helps not only to increase the selection intensity of parents but also to shorten the hybrid breeding cycle and produce higher genetic gains.

Furthermore, predicting two‐way and three‐way cross performance is of paramount importance in wheat as producing large numbers of hybrid combinations is not only costly but also impossible in some cases especially when the male and female parents do not have proper flowering nick. Moreover, the male parent needs to be an excellent pollen shedder in a given seed production environment to harvest the required amount of seed from sterile female plots. These limitations confine the hybrid testing program to a smaller set of parental lines, potentially leading to underutilization of all the diversity present within or among the breeding pools. On the other hand, with moderate to high prediction accuracies, genomic‐based prediction, as demonstrated in this study, may offer reliable estimates of hybrid performance from an unlimited number of crosses at a lower cost and within a shorter period.

### Variance Components of Models

Prediction accuracies of Models M1 through M5 for complex trait GY were not very different from the prediction accuracies of models including only pedigree × environment (M2) (0.64), genomic × environment (M4) (0.64), or both pedigree × environment together with genomic × environment (M5) (0.65). However, including both sources of interaction information is important for producing a consistent (but small) increase in the prediction accuracy of Model M5 explained by the clear increase in variance explained by the components resulting from genomic M_G (0.125) and M_G×E (0.077), as compared with those resulting from pedigree for M_A (0.085) and M_A×E (0.058) (Table 2). Therefore, although the increase in prediction accuracy may not seem very high, it is consistent and well explained by the magnitude of the genomic variance components of Model M5. Also, as previously shown, the male variance component resulting from genomic and pedigree in interactions with environments is sizeable in Models M1 through M4 for GY and also contributes to the relatively high prediction accuracy of these models.

### Environmental Covariables

Environmental covariables, especially average temperature, have significant influence on experimental yield potential from mid‐December to mid‐April (Lobell et al., 2005). In our study, Year 1 had the overall lowest yield (Fig. 2), probably as a result of higher minimum temperatures during tillering and grain filling (Supplemental Material—Environmental Covariables; http://hdl.handle.net/11529/10548129). Similarly, in Years 2 and 3, the overall minimum temperature during the growth period was lower than in Year 1, resulting in higher yield. However, total grain filling period in Year 3 was shorter than in Year 1, while early vigor and biomass were maintained through more tillers and increased plant height, which most probably helped to sustain the higher yield level in Year 3 than Year 1. The hybrids had an average yield advantage of 0.5 t ha^−1^ over the inbreds, which was comparable with some of the previous studies (Bedo et al., 2001; Cisar and Cooper, 2002). However, it was relatively lower than the yield advantage reported by Longin et al. (2013), who observed an average yield advantage of 1 t ha^−1^ or higher of hybrids than the inbred parents. This higher yield advantage of hybrids may be associated with the prolonged growing periods of winter wheat in Germany, which is 3 to 4 mo longer than the spring wheat growing period in Mexico. We did not observe substantial differences in average DTH and PHT between hybrids and parents, although we observed wide variation on an individual basis. Since major genes *Ppd‐1*, *Vrn‐1*, and *Rht‐1* are fixed in CIMMYT germplasm, the residual variation in DTH and PHT may be attributed to minor additive genes, which contribute to intermediate plant types in hybrids.

### Genomic‐Enabled Prediction Accuracy of the Models and Cross‐Validations

This study explores several prediction models under four types of cross‐validation schemes—T2FM, T1F, T1M, and T0FM—to address hybrid prediction problems in wheat. This is probably the first study where such dynamic prediction models are used on a large set of hybrid combinations produced from more than 600 parents. Hybrid prediction accuracy was highest for T2MF (where both males and females were observed and included in the training set in some environments) followed by T0MF (where neither parent was included in the training set). For the T2MF and T0MF validation schemes, the prediction accuracies for hybrid wheat yield were 0.62 to 0.65 and 0.46 to 0.61, respectively. These results were comparable with those of Zhao et al. (2015), who estimated accuracy as the correlation between predicted and observed phenotypic values standardized with the square root of heritability. However, these accuracies were higher than those previously reported by Zhao et al. (2013). A plausible explanation of T0FM's higher prediction accuracy is the larger number of parents used in the hybrid combinations and the robustness of models including pedigree and G×E. Compared with the study by Zhao et al. (2015), the number of parents we used to produce various hybrid combinations was nearly six times higher, which probably ensured that maximum genetic variation was captured in the training set, which in turn enabled us to predict the performance of the unobserved validation set more accurately.

Interestingly, the genomic‐enabled prediction accuracy of T0FM for the 18 males was indeed relatively high for several males for different traits and different models. For example, for GY, eight males had prediction accuracies higher than 0.60 for M5 (some of them achieving up to 0.8 accuracy, data not shown), whereas for other traits and models, a large number of males had high prediction accuracies reaching up to 0.8 to 0.9. As previously mentioned, these results can be explained by the large contribution to the total variance of the male genomic and pedigree additive variance and their interaction with environments. Also the high heritability of the trait GY and the other traits ensure high genomic‐enabled prediction accuracy

The predictability of the genomic main effect model (M1) is similar or better than that of the pedigree main effect model (M3) for cross‐validation schemes T2MF and T1F, whereas M3 performed better for T0MF. In general, prediction accuracy increased when interaction components were added to Models M3, M4, and M5. For GY, substantial increases in prediction accuracies were observed in cross‐validation schemes T1M and T0FM with G×E included, as compared with schemes without such interaction

The predictability of hybrids was higher in T1F than in T1M. This probably was due to the fact that a large number of crosses were made per female compared with fewer combinations per male, which means that the favorable genetic components in diverse females might have been left out of the training set. However, the addition of pedigree and G×E components improved the prediction accuracy significantly in T1M and T0MF. This result is in strong agreement with results previously report by Acosta‐Pech et al. (2017), who concluded that including G×E in the prediction of hybrid maize increased prediction accuracy significantly, that is, it was 12 to 22% higher than the prediction accuracy of models without G×E. This observation sheds lights on the importance of using pedigree or G×E when predicting unknown hybrid combinations, that is, performance of cross combination not evaluated in field trials. Knowing, a priori, which combination is likely to be successful is a key prediction problem in production of superior hybrids. The pedigree and G×E components appear to replenish the loss of information caused by missing parents in the training set.

Just like GY, similar trends in prediction accuracy were observed in other traits—DTH, DTF, DTM, and PHT—for different models and validation schemes. In agreement with previous studies, these prediction accuracies were largely driven by trait heritability, that is, higher heritability estimates were associated with higher prediction accuracy (Sukumaran et al., 2017; Zhang et al., 2017; Combs and Bernardo, 2013). In this study, the broad‐sense heritability estimates for DTH and DTF were high and comparable with those reported in previous studies (Zhao et al., 2015; 2013; Longin et al., 2013), whereas the heritability estimates for DTM and PHT were relatively lower as were the prediction accuracies. The low heritability of DTM as compared with DTH and DTF may be due to the complex nature of the grain‐filling phenomenon, nongenetic control, or higher G×E.

The prediction accuracy for GY ranged from 0.39 (M1 with T1M) to 0.65 (M5 with T2FM) across different models and CV schemes. The prediction accuracy increased by 0.11 to 0.14 by replacing the genomic main effect model (M1) with the genomic main effect and interaction model (M2) for GY, when prediction was performed on hybrids with an untested female (T1M) parent or with both parents untested (T0MF). Similar or greater improvements in prediction accuracy were observed when using the pedigree main effect model (M3) or interaction models (M4 and M5). Similar trends were observed for DTM and PHT, whereas very little increase in prediction accuracy was observed by changing models for DTH and DTF, suggesting that prediction accuracy is less dependent on models in traits with very high heritability estimates. The benefits of complex models are obvious in GY, the lower heritability trait. In addition to the genomic data, adding pedigree and G×E to the prediction model significantly enhances the prediction accuracy, especially when predicting the performance of hybrids that could be derived from untested parents.

## CONCLUSION

The results of this study show that a priori prediction of the best cross combination—the best combining male, female, or a specific combination between male and female—is possible using a complex model involving a pedigree‐based matrix, a marker‐based relationship matrix, and G×E. For GY—a complex trait with lower heritability—hybrid prediction can be helpful in accelerating the rate of genetic gain as well as for saving a high amount of financial resources for predicting hybrids that have never been evaluated in the field. Results of this study emphasize the importance of modeling GCA and SCA interacting with environments as a source for borrowing information existing not only among the males and females included in the hybrids but also for exploiting the genetic correlations among environments measured on all environmental covariables used to characterize the environments where the trials were performed. This study also demonstrates that genomic‐based hybrid prediction offers reliable predictions of hybrid performance from an unlimited number of crosses at a lower cost and in a shorter period.

### Supplemental Information Available

Supplemental information is available with the online version of this manuscript.

### Conflict of Interest Disclosure

The authors declare that there is no conflict of interest.

## Supporting information

Supplemental Information
Supplemental File 1
