# Supplementary material for: Hybrid Wheat Prediction Using Genomic, Pedigree, and Environmental Covariables Interaction Models
Source: Plant Genome. 2019 Mar 1;12(1):180051. doi: 10.3835/plantgenome2018.07.0051 (PMC12810112; doi:10.3835/plantgenome2018.07.0051)
Supplement: Supplementary file 1 [file TPG2-12-180051-s001.pdf]

SUPPLEMENTARY MATERIAL

Table S1. Prediction accuracy of 18 males for cross-validation T1M results for grain yield (GY).

Male	Model				
	M1	M2	M3	M4	M5
4313703	0.3867	0.4318	0.3952	0.4671	0.4526
4755014	0.5306	0.5787	0.5263	0.5646	0.6025
5996524	0.4459	0.4769	0.4096	0.4227	0.4637
5995337	0.3649	0.3655	0.2914	0.1854	0.4425
6001014	0.5852	0.6309	0.5475	0.6157	0.6251
6001072	0.2816	0.2807	0.4229	0.4198	0.3146
6279600	--	--	--	--	--
6280120	--	--	--	--	--
5895423	0.5061	0.5597	0.5713	0.6457	0.6079
5994020	0.6846	0.7191	0.6886	0.7342	0.7339
6001016	0.3170	0.3341	0.3558	0.3427	0.3453
6176013	0.7089	0.7369	0.7088	0.694	0.7433
5999970	0.4899	0.5105	0.4095	0.4136	0.4603
5995610	0.6622	0.7631	0.6824	0.6017	0.6681
6417352	0.0828	0.2438	0.6652	0.3049	0.2985
7456108	0.5048	0.6370	0.4435	0.7377	0.6740
7810933	0.2177	0.1164	0.2570	0.1102	0.0525
6417117	-0.4607	0.5998	0.3669	0.7074	0.6932

Table S2. Prediction accuracy of 18 males for cross-validation T1M results for days to heading (DTH).

Male	Model				
	M1	M2	M3	M4	M5
4313703	0.7555	0.7650	0.7598	0.7696	0.7660
4755014	0.8182	0.8388	0.8360	0.8471	0.8530
5996524	0.7585	0.7877	0.7731	0.7924	0.792
5995337	0.7124	0.7087	0.6566	0.6386	0.6774
6001014	0.8478	0.8404	0.8455	0.8432	0.8483
6001072	0.6444	0.6142	0.6661	0.6330	0.6251
6279600	--	--	--	--	--
6280120	--	--	--	--	--
5895423	0.7862	0.7944	0.7944	0.7958	0.8015
5994020	0.8352	0.8446	0.8469	0.8491	0.8555
6001016	0.8874	0.8845	0.8741	0.8829	0.8859
6176013	0.7675	0.7768	0.8104	0.8193	0.7908
5999970	0.8593	0.8734	0.8630	0.8770	0.8793
5995610	0.7447	0.5693	0.7686	0.6590	0.5766
6417352	0.5679	0.5769	0.6160	0.6105	0.6039
7456108	0.8561	0.7974	0.8641	0.8210	0.7998
7810933	0.9507	0.9245	0.9393	0.9178	0.9285
6417117	-0.6686	-0.7883	-0.6111	-0.7669	-0.7259

Table S3. Prediction accuracy of 18 males for cross-validation T1M results for days to flowering (DTF).

Male	Model				
	M1	M2	M3	M4	M5
4313703	0.7526	0.7628	0.7567	0.7593	0.7662
4755014	0.8091	0.8330	0.8279	0.8396	0.8446
5996524	0.7465	0.7724	0.7616	0.7811	0.7774
5995337	0.8615	0.8407	0.8059	0.7844	0.8141
6001014	0.8358	0.8246	0.8350	0.8342	0.8386
6001072	0.6014	0.5998	0.6490	0.6312	0.6037
6279600	--	--	--	--	--
6280120	--	--	--	--	--
5895423	0.7824	0.7927	0.7904	0.7911	0.8007
5994020	0.8383	0.8483	0.8486	0.8512	0.8584
6001016	0.8814	0.8740	0.8638	0.8686	0.8782
6176013	0.7623	0.7681	0.8062	0.8165	0.7933
5999970	0.8447	0.8631	0.8477	0.8645	0.864
5995610	0.7370	0.5526	0.7999	0.6601	0.5807
6417352	0.5130	0.6149	0.5659	0.5974	0.6623
7456108	0.8524	0.7481	0.8185	0.7417	0.7974
7810933	0.9473	0.9449	0.9155	0.8889	0.9496
6417117	-0.7731	-0.757	-0.6598	-0.7275	-0.7676

Table S4. Prediction accuracy of 18 males for cross-validation T1M results for days to maturity (DTM).

Parent	Model				
	M1	M2	M3	M4	M5
4313703	0.4330	0.5167	0.4940	0.5466	0.5342
4755014	0.5065	0.6570	0.5955	0.6942	0.6985
5996524	0.4496	0.6092	0.4848	0.6208	0.6209
5995337	0.6359	0.5948	0.6765	0.7734	0.7177

6001014	0.6898	0.7444	0.6894	0.7419	0.7618
6001072	0.4857	0.5456	0.5379	0.5573	0.5713
6279600	--	--	--	--	--
6280120	--	--	--	--	--
5895423	0.5495	0.6219	0.5798	0.6310	0.6311
5994020	0.5769	0.6267	0.6012	0.6561	0.6585
6001016	0.5154	0.5403	0.4484	0.4965	0.5334
6176013	0.2803	0.2863	0.2972	0.4035	0.3451
5999970	0.6790	0.7125	0.6570	0.6722	0.6911
5995610	0.7787	0.9443	0.7158	0.9118	0.9393
6417352	0.2629	0.6006	0.1162	0.3749	0.4187
7456108	-0.6400	-0.6612	-0.3897	-0.4946	-0.6376
7810933	-0.3367	-0.5385	0.4801	0.5292	-0.3097
6417117	0.6983	0.5890	0.6133	0.5129	0.5884

Table S5. Prediction accuracy of 18 males for cross-validation T1M results for plant height (PHT).

Male	Model				
	M1	M2	M3	M4	M5
4313703	0.3736	0.4555	0.381	0.4672	0.4684
4755014	0.4958	0.5648	0.5077	0.5728	0.5889
5996524	0.3974	0.4512	0.398	0.4425	0.4584
5995337	0.4218	0.3130	0.3682	0.4691	0.4112
6001014	0.5454	0.6019	0.5878	0.6327	0.6107
6001072	0.5000	0.5334	0.4518	0.4958	0.5237
6279600	--	--	--	--	--
6280120	--	--	--	--	--
5895423	0.5708	0.6699	0.5547	0.6269	0.6718
5994020	0.6127	0.6817	0.5075	0.598	0.6637
6001016	0.7207	0.7122	0.6811	0.7251	0.7148
6176013	0.3706	0.6291	0.2556	0.4716	0.5769

5999970	0.4543	0.5998	0.587	0.6618	0.6332
5995610	0.5122	0.9457	0.4316	0.8577	0.9311
6417352	-0.1798	0.4396	0.0111	0.5978	0.5335
7456108	0.2328	0.8329	-0.1277	0.6803	0.7912
7810933	0.5949	0.8091	0.0371	0.4322	0.7680
6417117	0.1337	0.0958	0.6649	0.5168	0.18

Table S6. Prediction accuracy of 18 males for cross-validation T0FM results for grain yield (GY).

Male	Model				
	M1	M2	M3	M4	M5
4313703	0.457	0.4814	0.4806	0.5288	0.5059
4755014	0.5497	0.5773	0.5079	0.5429	0.5870
5996524	0.4467	0.4535	0.4008	0.4130	0.4517
5995337	0.6170	0.5642	0.5619	0.2285	0.5702
6001014	0.6348	0.6951	0.6353	0.6968	0.6799
6001072	0.2524	0.2444	0.4431	0.4381	0.3170
6279600	0.8101	0.6312	0.7124	0.5576	0.5806
6280120	0.3696	0.440	0.4075	0.4429	0.4331
5895423	0.5222	0.5209	0.6242	0.6449	0.6085
5994020	0.7015	0.7250	0.6763	0.7073	0.7310
6001016	0.2169	0.2169	0.1879	0.1858	0.2343
6176013	0.6922	0.7174	0.6895	0.6849	0.7155
5999970	0.4554	0.4846	0.4931	0.4999	0.5245
5995610	0.5812	0.6238	0.4461	0.5898	0.5349
6417352	0.3207	0.9835	0.9987	0.7622	0.8194
7456108	0.8432	0.9399	0.9482	0.9797	0.9428
7810933	0.6704	0.8885	0.9555	0.8650	0.9187
6417117	-0.8223	0.5904	0.3515	0.6935	0.7494

Table S7. Prediction accuracy of 18 males for cross-validation T0FM results for days to heading (DTH).

Male	Model				
	M1	M2	M3	M4	M5
4313703	0.7503	0.7526	0.7464	0.7524	0.7587
4755014	0.8358	0.8461	0.8319	0.8383	0.8518
5996524	0.7792	0.7994	0.7955	0.8068	0.8069
5995337	0.6642	0.6683	0.6249	0.6388	0.6601
6001014	0.7106	0.7022	0.7441	0.7055	0.7357
6001072	0.8142	0.7119	0.7383	0.7566	0.7257
6279600	0.4392	0.3648	0.4001	0.3662	0.3588
6280120	0.2814	0.2509	0.1887	0.1572	0.2168
5895423	0.7606	0.7642	0.7732	0.7646	0.7746
5994020	0.8499	0.8519	0.8335	0.8375	0.8456
6001016	0.9024	0.8974	0.8853	0.8886	0.8978
6176013	0.7295	0.7165	0.8007	0.7858	0.7423
5999970	0.8083	0.8376	0.8145	0.8324	0.8376
5995610	0.9485	0.9075	0.9455	0.9475	0.9125
6417352	0.6137	0.8562	0.6225	0.7205	0.7586
7456108	0.9977	0.9570	0.9928	0.9918	0.999
7810933	0.9759	0.9539	0.9615	0.9678	0.9665
6417117	-0.3842	-0.1781	-0.4616	-0.582	-0.3633

Table S8. Prediction accuracy of 18 males for cross-validation T0FM results for days to flowering (DTF).

Male	Model				
	M1	M2	M3	M4	M5
4313703	0.7424	0.7441	0.7404	0.7516	0.7479
4755014	0.8262	0.8366	0.8245	0.8327	0.8472
5996524	0.7666	0.7878	0.7836	0.7942	0.7949
5995337	0.8976	0.9018	0.8645	0.8818	0.8998
6001014	0.6974	0.6960	0.7386	0.7250	0.7354
6001072	0.7039	0.7042	0.7403	0.7455	0.7159
6279600	0.4849	0.3973	0.4094	0.4073	0.4142
6280120	0.3603	0.3542	0.2650	0.2665	0.3431
5895423	0.7571	0.7640	0.7677	0.7649	0.7733
5994020	0.8512	0.8564	0.8355	0.8412	0.8508
6001016	0.8911	0.8827	0.8706	0.8731	0.8844
6176013	0.7312	0.7171	0.7903	0.7786	0.7465
5999970	0.7831	0.8111	0.7933	0.8115	0.8166
5995610	0.9548	0.8943	0.9526	0.9266	0.9048
6417352	0.4912	0.8257	0.6217	0.8930	0.8444
7456108	0.9994	0.9928	0.9793	0.9979	0.9192
7810933	0.9556	0.9546	0.9615	0.9734	0.9595
6417117	-0.5507	0.0726	-0.5659	-0.6135	-0.5080

Table S9. Prediction accuracy of 18 males for cross-validation T0FM results for days to maturity (DTM).

Male	Model				
	M1	M2	M3	M4	M5
4313703	0.4684	0.5340	0.5285	0.5996	0.571
4755014	0.5562	0.6663	0.5814	0.6939	0.7048
5996524	0.5022	0.6282	0.5483	0.6503	0.6455
5995337	0.7003	0.6151	0.7245	0.8293	0.8714

6001014	0.5978	0.6209	0.6404	0.6118	0.6353
6001072	0.5980	0.6157	0.6341	0.6147	0.6277
6279600	-0.0924	0.2875	0.0159	0.2542	0.2981
6280120	0.2753	0.1457	0.1591	0.2169	0.1954
5895423	0.5608	0.6635	0.5927	0.6537	0.6697
5994020	0.6062	0.6526	0.6101	0.6462	0.6748
6001016	0.5749	0.6172	0.4907	0.5444	0.5917
6176013	0.3629	0.4780	0.3841	0.4318	0.466
5999970	0.7015	0.6839	0.6371	0.6386	0.6586
5995610	0.8164	0.9467	0.6201	0.8896	0.9101
6417352	-0.8817	-0.5826	-0.9119	-0.4798	-0.4634
7456108	0.0505	0.0237	0.8220	0.7043	0.1776
7810933	0.7626	0.7272	0.7554	0.7801	0.6317
6417117	0.4108	-0.8874	0.0811	-0.6915	-0.7704

Table S10. Prediction accuracy of 18 males for cross-validation T0FM results for plant height (PHT).

Male	Model				
	M1	M2	M3	M4	M5
4313703	0.3983	0.4838	0.3760	0.4565	0.4940
4755014	0.4968	0.5281	0.4919	0.5235	0.5434
5996524	0.4423	0.4943	0.4511	0.4768	0.5046
5995337	0.4018	0.2290	0.5047	0.5886	0.5485
6001014	0.4503	0.4928	0.4827	0.5493	0.5100
6001072	0.5154	0.5986	0.4712	0.5338	0.6018
6279600	0.5257	0.5346	0.5513	0.5486	0.5532
6280120	0.5849	0.4358	0.4341	0.3310	0.4646
5895423	0.5282	0.5940	0.4858	0.5650	0.6020
5994020	0.5837	0.6739	0.4344	0.5394	0.6181
6001016	0.8327	0.8325	0.7752	0.8123	0.8306
6176013	0.3005	0.5775	0.2567	0.5473	0.5573

5999970	0.4981	0.6381	0.6061	0.6934	0.6531
5995610	0.7991	0.9617	0.7558	0.9873	0.9885
6417352	-0.5284	0.9088	-0.3255	0.8606	0.9996
7456108	0.7178	0.7674	0.8163	0.5823	0.7013
7810933	0.7833	0.8399	0.6927	0.9378	0.8820
6417117	-0.8516	-0.9455	0.1477	-0.94	-0.9090

SUPPLEMENTARY MATERIAL

Scripts for fitting the models using BGLR

This section contains supplementary R code for fitting the models presented in this study. The models were fitted using the BGLR statistical package (de los Campos and Pérez-Rodríguez, 2016). We use the same computational approach described in Acosta-Pech et al., 2017 to speed up computations, in BGLR, we use the eigen-value decomposition of the variance-covariance matrices in all models. Let $\boldsymbol{Zu}$, $\boldsymbol{u} \sim \boldsymbol{N}(\boldsymbol{0}, \sigma_{\mathrm{a}}^2 \boldsymbol{A})$, with $\boldsymbol{A}$ a variance-covariance matrix and σ_{a}^2 a variance component, let $\boldsymbol{\Gamma \Lambda \Gamma}' = \boldsymbol{A}$, with $\boldsymbol{\Gamma}$ the eigen-vectors of $\boldsymbol{A}$ and $\boldsymbol{\Lambda}$ its corresponding eigen-values; then $\boldsymbol{Zu} \stackrel{d}{=} \boldsymbol{Z\Gamma\Lambda}^{\frac{1}{2}}\boldsymbol{u}^*$, where $\boldsymbol{u}^* \sim \boldsymbol{N}(\boldsymbol{0}, \sigma_{\mathrm{a}}^2 \boldsymbol{I})$ and "$\stackrel{d}{=}$" stands for equality in distribution. By using this result, model $\boldsymbol{y} = \boldsymbol{Zu} + \boldsymbol{\varepsilon}$ can be written as $\boldsymbol{y} = \boldsymbol{Z\Gamma\Lambda}^{\frac{1}{2}}\boldsymbol{u}^* + \boldsymbol{\varepsilon}$, which is known as Bayesian Ridge Regression (BRR), a model that can be fitted very efficiently in the BGLR software-package. All the models were rewritten using the representation described above. Below you will find the description of the data objects and the R codes needed to fit the models.

Description of Data Objects

- G1: A genomic additive relationship matrix for males. The row.names/column.names of G1 corresponds to the ids of the males.
- G1: A genomic additive relationship matrix for females. The row.names/column.names of G2 correspond to the ids of the females.
- A1: Additive relationship matrix derived from pedigree for males. The row.names/column.names of A1 corresponds to the ids of the males.
- A2: Additive relationship matrix derived from pedigree for females. The row.names/column.names of A2 corresponds to the ids of the females.
- Pheno: A data.frame with 4 columns:
    - y: Response variable.
    - P1: ids for males.
    - P2: ids for females.
    - Year: ids for years (Environment).

Genomic/Pedigree relationship and incidence matrices

- **H_G** (Relationship between hybrids using relationships derived from markers)

```
P1=rownames(G1)

P2=rownames(G2)


#Unique hybrids

hybrid=paste(Pheno$P1, Pheno$P2,sep="@")

hybrid=unique(hybrid)


#Build the relationship matrix for hybrids using markers

nh=length(hybrid)

H_G=matrix(NA,nrow=nh,ncol=nh)


for(k in 1:nh)

{

    for(l in 1:nh)

    {

        tmp1=strsplit(hybrid[k],"@")[[1]]

        tmp2=strsplit(hybrid[l],"@")[[1]]

        i=which(P1==tmp1[1])

        istar=which(P1==tmp2[1])

        j=which(P2==tmp1[2])

        jstar=which(P2==tmp2[2])

        H_G[k,l]=G1[i,istar]*G2[j,jstar]

    }

}
```

```
rownames(H_G)=colnames(H_G)=hybrid


#Make P1 a factor and order the rows and columns of G1 accordingly

P1=as.factor(P1)

index=order(P1)

G1=G1[index,index]


#Make P2 a factor and order the rows and columns of G2 accordingly

P2=as.factor(P2)

index=order(P2)

G2=G2[index,index]
```

- **H_A** (Relationship between hybrids using relationships derived from pedigree)

```
P1=rownames(A1)

P2=rownames(A2)


H_A=matrix(NA,nrow=nh,ncol=nh)


for(k in 1:nh)

{

    for(l in 1:nh)

    {

        tmp1=strsplit(hybrid[k],"@")[[1]]

        tmp2=strsplit(hybrid[l],"@")[[1]]

        i=which(P1==tmp1[1])

        istar=which(P1==tmp2[1])

        j=which(P2==tmp1[2])
```

```
            jstar=which(P2==tmp2[2])

            H_A[k,l]=A1[i,istar]*A2[j,jstar]

      }

}


rownames(H_A)=colnames(H_A)=hybrid


#Make P1 a factor and order the rows and columns of G1 accordingly

P1=as.factor(P1)

index=order(P1)

A1=A1[index,index]


#Make P2 a factor and order the rows and columns of G2 accordingly

P2=as.factor(P2)

index=order(P2)

A2=A2[index,index]


#Order rows and columns of H_G and H_A


#Make hybrid a factor and order the rows and columns of H accordingly

hybrid=as.factor(hybrid)

index=order(hybrid)

H_G=H_G[index,index]

H_A=H_A[index,index]
```

- Incidence matrices $\boldsymbol{Z_E}, \boldsymbol{Z_M}, \boldsymbol{Z_F}, \boldsymbol{Z_H}$ and response variable

```
hybrid=as.factor(paste(Pheno$P1,Pheno$P2,sep="@"))

Loc=as.factor(Pheno$Year)

index=order(Loc,hybrid)

Pheno=Pheno[index,]

hybrid=hybrid[index]

Loc=Loc[index]


P1=as.factor(Pheno$P1)

P2=as.factor(Pheno$P2)


#Response variable

y=Pheno$yield_blue


#Incidence matrices

Z1=model.matrix(~P1-1) #Z for males

Z2=model.matrix(~P2-1) #Z for females

Zh=model.matrix(~hybrid-1) #Z for hybrids

ZE=model.matrix(~Loc-1)    #Z for environment

ZE_ZEt=tcrossprod(ZE)


hybrid=as.factor(paste(Pheno$P1,Pheno$P2,sep="@"))

Loc=as.factor(Pheno$Loc)

index=order(Loc,hybrid)

Pheno=Pheno[index,]
```

```
hybrid=hybrid[index]
Loc=Loc[index]

P1=as.factor(Pheno$P1)
P2=as.factor(Pheno$P2)

#Response variable
y=Pheno$y

#Incidence matrices
Z_D=model.matrix(~P1-1)
Z_F=model.matrix(~P2-1)
Z_h=model.matrix(~hybrid-1)
Z_E=model.matrix(~Loc-1)
Z_E_Z_Et=tcrossprod(Z_E)

#Eigen-value decompositions to speed up computations
EVD_G1=eigen(G1)
index=EVD_G1$values>1e-10
EVD_G1$vectors=EVD_G1$vectors[,index]
EVD_G1$values=EVD_G1$values[index]

EVD_A1=eigen(A1)
index=EVD_A1$values>1e-10
EVD_A1$vectors=EVD_A1$vectors[,index]
```

```
EVD_A1$values=EVD_A1$values[index]

EVD_G2=eigen(G2)
index=EVD_G2$values>1e-10
EVD_G2$vectors=EVD_G2$vectors[,index]
EVD_G2$values=EVD_G2$values[index]

EVD_A2=eigen(A2)
index=EVD_A2$values>1e-10
EVD_A2$vectors=EVD_A2$vectors[,index]
EVD_A2$values=EVD_A2$values[index]

EVD_H_G=eigen(H_G)
index=EVD_H_G$values>1e-10
EVD_H_G$vectors=EVD_H_G$vectors[,index]
EVD_H_G$values=EVD_H_G$values[index]

EVD_H_A=eigen(H_A)
index=EVD_H_A$values>1e-10
EVD_H_A$vectors=EVD_H_A$vectors[,index]
EVD_H_A$values=EVD_H_A$values[index]

Z1_Gstar=Z1%*%EVD_G1$vectors%*%sqrt(diag(EVD_G1$values))
Z2_Gstar=Z2%*%EVD_G2$vectors%*%sqrt(diag(EVD_G2$values))
Zh_Gstar=Zh%*%EVD_H_G$vectors%*%sqrt(diag(EVD_H_G$values))
```

```
Z1_Astar=Z1%*%EVD_A1$vectors%*%sqrt(diag(EVD_A1$values))

Z2_Astar=Z2%*%EVD_A2$vectors%*%sqrt(diag(EVD_A2$values))

Zh_Astar=Zh%*%EVD_H_A$vectors%*%sqrt(diag(EVD_H_A$values))


#Linear predictor for model 1 based on markers


ETA1=list(list(X=ZE,model="BRR"),

    list(X=Z1_Gstar,model="BRR"),

    list(X=Z2_Gstar,model="BRR"),

    list(X=Zh_Gstar,model="BRR"))


#Linear predictor for model 1 based on pedigree

ETA2=list(list(X=ZE,model="BRR"),

    list(X=Z1_Astar,model="BRR"),

    list(X=Z2_Astar,model="BRR"),

    list(X=Zh_Astar,model="BRR"))



#Additional incidence matrices

Vh_G=tcrossprod(Zh_Gstar)*ZE_ZEt

EVD_Vh_G=eigen(Vh_G)

index=EVD_Vh_G$values>1e-10

EVD_Vh_G$vectors=EVD_Vh_G$vectors[,index]

EVD_Vh_G$values=EVD_Vh_G$values[index]
```

```
ZhGxEstar=EVD_Vh_G$vectors%*%sqrt(diag(EVD_Vh_G$values))


Vh_A=tcrossprod(Zh_Astar)*ZE_ZEt

EVD_Vh_A=eigen(Vh_A)

index=EVD_Vh_A$values>1e-10

EVD_Vh_A$vectors=EVD_Vh_A$vectors[,index]

EVD_Vh_A$values=EVD_Vh_A$values[index]


ZhAxEstar=EVD_Vh_A$vectors%*%sqrt(diag(EVD_Vh_A$values))


V1_G=tcrossprod(Z1_Gstar)*ZE_ZEt

EVD_V1_G=eigen(V1_G)

index=EVD_V1_G$values>1e-10

EVD_V1_G$vectors=EVD_V1_G$vectors[,index]

EVD_V1_G$values=EVD_V1_G$values[index]

Z1_GxEstar=EVD_V1_G$vectors%*%sqrt(diag(EVD_V1_G$values))


V1_A=tcrossprod(Z1_Astar)*ZE_ZEt

EVD_V1_A=eigen(V1_A)

index=EVD_V1_A$values>1e-10

EVD_V1_A$vectors=EVD_V1_A$vectors[,index]

EVD_V1_A$values=EVD_V1_A$values[index]

Z1_AxEstar=EVD_V1_A$vectors%*%sqrt(diag(EVD_V1_A$values))
```

```
V2_G=tcrossprod(Z2_Gstar)*ZE_ZEt

EVD_V2_G=eigen(V2_G)

index=EVD_V2_G$values>1e-10

EVD_V2_G$vectors=EVD_V2_G$vectors[,index]

EVD_V2_G$values=EVD_V2_G$values[index]

Z2_GxEstar=EVD_V2_G$vectors%*%sqrt(diag(EVD_V2_G$values))


V2_A=tcrossprod(Z2_Astar)*ZE_ZEt

EVD_V2_A=eigen(V2_A)

index=EVD_V2_A$values>1e-10

EVD_V2_A$vectors=EVD_V2_A$vectors[,index]

EVD_V2_A$values=EVD_V2_A$values[index]

Z2_AxEstar=EVD_V2_A$vectors%*%sqrt(diag(EVD_V2_A$values))


#Linear predictor

ETA3=list(list(X=ZE,model="BRR"),

    list(X=Z1_Gstar,model="BRR"),

    list(X=Z2_Gstar,model="BRR"),

    list(X=Zh_Gstar,model="BRR"),

    list(X=ZhGxEstar,model="BRR"),

    list(X=Z1_GxEstar,model="BRR"),

    list(X=Z2_GxEstar,model="BRR"))



#Linear predictor
```

```
ETA4=list(list(X=ZE,model="BRR"),
     list(X=Z1_Astar,model="BRR"),
     list(X=Z2_Astar,model="BRR"),
     list(X=Zh_Astar,model="BRR"),
     list(X=ZhAxEstar,model="BRR"),
     list(X=Z1_AxEstar,model="BRR"),
     list(X=Z2_AxEstar,model="BRR"))



ETA5=list(list(X=ZE,model="BRR"),
                 list(X=Z1_Gstar,model="BRR"),
                 list(X=Z2_Gstar,model="BRR"),
                list(X=Zh_Gstar,model="BRR"),
                list(X=ZhGxEstar,model="BRR"),
                list(X=Z1_GxEstar,model="BRR"),
                list(X=Z2_GxEstar,model="BRR"),
                list(X=Z1_Astar,model="BRR"),
                list(X=Z2_Astar,model="BRR"),
                list(X=Zh_Astar,model="BRR"),
                list(X=ZhAxEstar,model="BRR"),
                list(X=Z1_AxEstar,model="BRR"),
                list(X=Z2_AxEstar,model="BRR"))
```

Fitting the models

Fitting Model 1:

```
library(BGLR)
fm1=BGLR(y=y,ETA=ETA1,nIter=50000,burnIn=25000)
```

Fitting Model 2:

```
library(BGLR)
fm2=BGLR(y=y,ETA=ETA2,nIter=50000,burnIn=25000)
```

Fitting Model 3:

```
library(BGLR)
fm3=BGLR(y=y,ETA=ETA3,nIter=50000,burnIn=25000)
```

Fitting Model 4:

```
library(BGLR)
fm4=BGLR(y=y,ETA=ETA4,nIter=50000,burnIn=25000)
```

Fitting Model 5:

```
library(BGLR)
fm5=BGLR(y=y,ETA=ETA5,nIter=50000,burnIn=25000)
```
